# High-Grade Solid Adenoid Cystic Carcinoma of the Larynx: A Case Report

**DOI:** 10.3390/reports8040195

**Published:** 2025-10-01

**Authors:** Matteo Aldo Russo, Stefano Patruno, Christian Fiorentino, Pietro Corsa, Teodoro Aragona, Lucia Maria Dimitri, Michele Cassano, Lazzaro Cassano

**Affiliations:** 1 Department of Maxillofacial Surgery and Otolaryngology, IRCCS Hospital Casa Sollievo della Sofferenza, 71013 San Giovanni Rotondo, Foggia, Italy; m.russo@operapadrepio.it (M.A.R.); t.aragona@operapadrepio.it (T.A.); l.cassano@operapadrepio.it (L.C.); 2 Department of Otorhinolaryngology-Head and Neck Surgery, University Hospital of Foggia, 71100 Foggia, Italy; stefpat444@gmail.com (S.P.); michele.cassano@unifg.it (M.C.); 3 Department of Radiotherapy, IRCCS Hospital Casa Sollievo della Sofferenza, 71013 San Giovanni Rotondo, Foggia, Italy; p.corsa@operapadrepio.it; 4 Department of Pathological Anatomy, IRCCS Hospital Casa Sollievo della Sofferenza, 71013 San Giovanni Rotondo, Foggia, Italy; l.dimitri@operapdrepio.it

**Keywords:** adenoid cystic carcinoma, larynx, treatment, radiotherapy, surgery, management

## Abstract

**Background and Clinical Significance**: Adenoid cystic carcinoma (ACC) is a rare neoplasm of salivary glands, accounting for approximately 2–4% of all ACCs of head and neck malignancies. Adenoid cystic carcinoma (ACC) of the larynx is exceedingly rare, accounting for only 0.07–0.25% of all laryngeal tumors. Within the larynx, ACC may arise in various locations; however, the subglottic region is most commonly affected, representing approximately 64% of cases. ACC typically manifests as a slow-growing tumor with a pronounced tendency for perineural invasion and local recurrence. Current treatment strategies primarily involve surgical resection followed by adjuvant radiotherapy. Chemotherapy demonstrates limited efficacy and is generally reserved for advanced, recurrent, or metastatic disease. Given the rarity of this malignancy and the limited number of cases reported in the literature, we aim to contribute to the existing body of knowledge by presenting a clinical case of laryngeal ACC. **Case Presentation**: A 77-year-old male with a significant smoking history (more than 20 cigarettes per day for over 40 years) presented to our department in October 2023 with persistent dysphonia lasting several months. Endoscopic evaluation of the upper aerodigestive tract revealed an extensive neoplastic lesion involving the larynx. Contrast-enhanced computed tomography (CT) confirmed the presence and extent of the lesion. The patient subsequently underwent surgical resection and was referred for adjuvant postoperative radiotherapy. Unfortunately, the patient died of a myocardial infarction a few days before radiotherapy could be initiated. **Conclusions**: Due to the rarity of laryngeal adenoid cystic carcinoma, further studies are necessary to define optimal management strategies. Sharing clinical experiences and outcomes is essential, as there is currently no universally accepted treatment consensus for this uncommon malignancy. At the same time, our aim is to highlight the importance of histological subtype and perineural invasion which have to be considered as important prognostic factors when dealing with ACC.

## 1. Introduction and Clinical Significance

Malignant neoplasms of the larynx can be classified into two main categories: squamous cell carcinoma (SCC) and non-squamous cell carcinoma (non-SCC), with the latter accounting for approximately 10% of cases. Among non-SCCs, adenoid cystic carcinoma (ACC) is a rare malignant tumor of the larynx. ACC constitutes approximately 7.5–10% of all salivary gland neoplasms and can arise in both major and minor salivary glands [1,2,3]. Laryngeal involvement by ACC is extremely rare, representing only 0.07–0.25% of all laryngeal tumors. It most commonly affects the subglottic region due to the predominance of minor salivary glands in this area; however, it may also occur in other locations, including the supraglottic region, glottic plane, aryepiglottic folds, and caudal portion of the epiglottis [1,4,5,6,7]. Laryngeal ACC typically presents between the fifth and seventh decades of life, with no gender predilection [1]. Notably, smoking is not considered a risk factor, and its etiology remains unclear [5,8,9,10,11]. Diagnosis is often delayed due to the tumor’s indolent growth, submucosal spread, and frequent subglottic involvement [9,12]. ACC is characterized by a high propensity for perineural invasion and distant metastasis—most commonly in the lungs, liver, and bones—which occur in 30–50% of cases. Lymph node involvement is uncommon, with the lungs being the most frequent site of metastasis [1,8,12,13,14,15,16]. Clinical presentation varies with tumor location [5,8,10,11,17]. Due to the rarity of laryngeal ACC, standardized treatment guidelines are currently lacking; therefore, management is primarily based on expert opinion [12,18]. Each reported case contributes valuable clinical insight into this uncommon entity. In this report, we describe our experience in diagnosing and managing a rare case of laryngeal adenoid cystic carcinoma (Table 1).

## 2. Case Presentation

**Figure 1:**
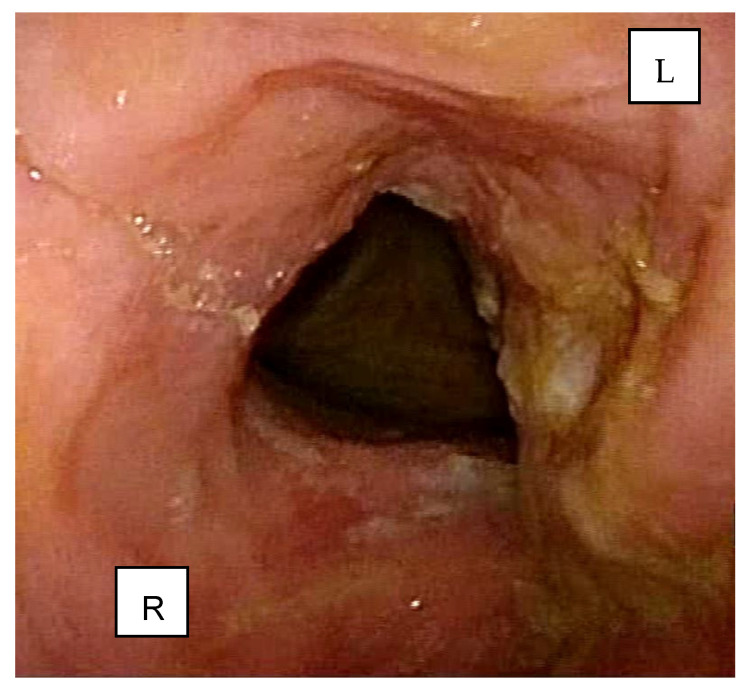
Endoscopic evaluation showing an extensive neoformation involving both true vocal cords, the left laryngeal ventricle, and the left false vocal cord.

A 77-year-old male with a history of heavy smoking (more than 20 cigarettes per day for over 40 years) presented in October 2023 with persistent dysphonia lasting several months. Otolaryngologic examination, including endoscopic evaluation of the upper aerodigestive tract, revealed an extensive neoformation involving both true vocal cords, the left laryngeal ventricle, and the left false vocal cord (Figure 1). Hypomobility was observed in the left vocal fold and the ipsilateral cricoarytenoid unit.

**Figure 2:**
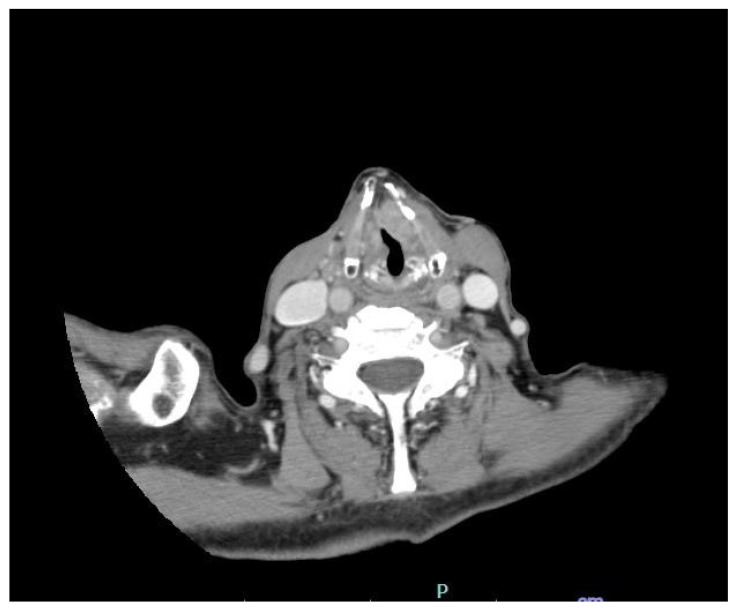
CT Scan demonstrated irregularity of the anterior glottic contour with involvement of the anterior commissure and contrast enhancement of the mucosa and submucosa, along with obliteration of the paralaryngeal fat planes.

**Diagnostic Workup:** The patient was advised to undergo further diagnostic evaluation and was subsequently admitted to our institution, Casa Sollievo della Sofferenza Hospital, in San Giovanni Rotondo a few days later. Repeat endoscopic assessment confirmed the initial findings; therefore, a contrast-enhanced computed tomography (CT) scan was performed to evaluate the extent of the lesion and assess for lymph node involvement. The scan demonstrated irregularity of the anterior glottic contour, with involvement of the anterior commissure, contrast enhancement of the mucosa and submucosa, and obliteration of the paralaryngeal fat planes (Figure 2).

**Figure 3:**
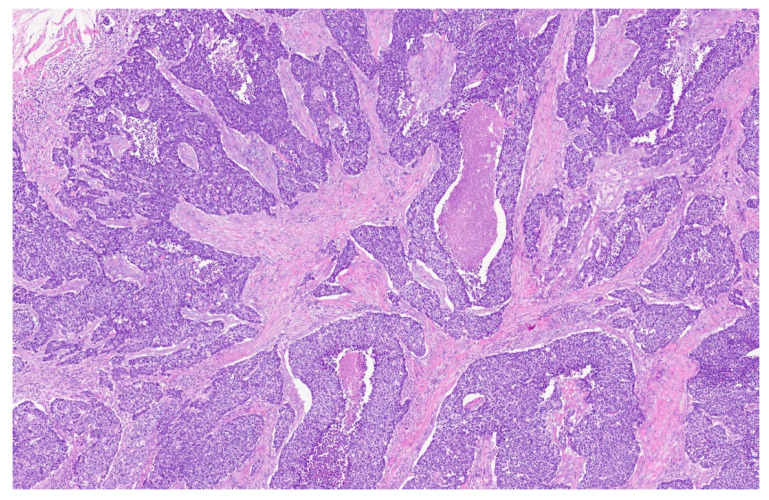
Histological section showing the solid pattern with foci of necrosis (7×).

**Figure 4:**
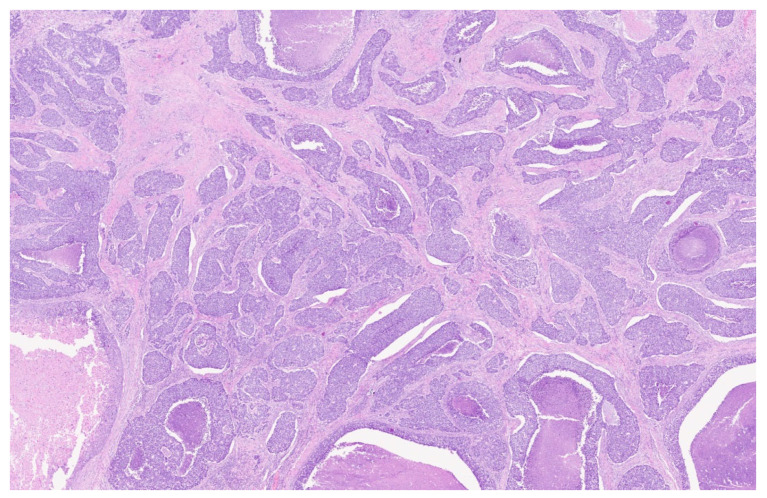
Histological sections showing the solid pattern with foci of necrosis (3×).

No overt lymphadenopathy was detected; however, a suspicious subcentimetric lymph node was identified at level III on the left side of the neck. **Histological Diagnosis:** Based on these findings, a few days later the patient underwent microlaryngoscopy with biopsy. Histopathological examination revealed adenoid cystic carcinoma (ACC) of the minor salivary glands of the larynx, solid variant. The clinical TNM (cTNM) defined, according to the results of these investigations, was T3 N1 M0. Two days later this case was then discussed by multidisciplinary tumor board—comprising an otolaryngologist, medical oncologist, radiation oncologist, radiologist, and pathologist— which recommended surgical intervention followed by adjuvant radiotherapy. While discussing this case, our multidisciplinary tumor board also performed a review of the available literature, published within the last ten years, on this topic on Pub-Med, using as a keyword “adenoid cystic carcinoma of larynx”. A table summarizing the results is presented for the readers, to provide a focus on the most recent papers available in the literature on ACC (Table 2). **Surgical Treatment:** Within ten days the patient underwent total laryngectomy with bilateral selective neck dissection (levels II, III, and IV). A tracheoesophageal voice prosthesis was placed. OPHL was not considered as a possible treatment option, due to the age of the patient. Histological examination of the surgical specimen confirmed a poorly differentiated, high-grade carcinoma, characterized by solid and comedonecrotic growth patterns, consistent with adenoid cystic carcinoma of the larynx, as defined by the 2017 WHO classification (Figure 3 and Figure 4).

**Figure 5:**
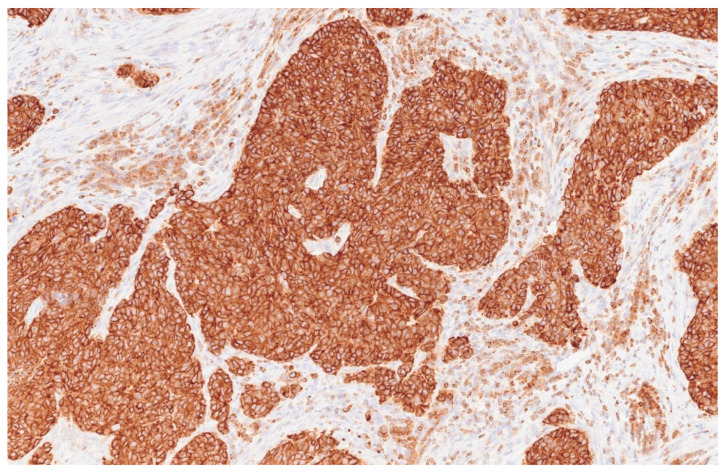
Adenoid cystic carcinoma section: Anti-Bcl2 antibody (20×).

**Figure 6:**
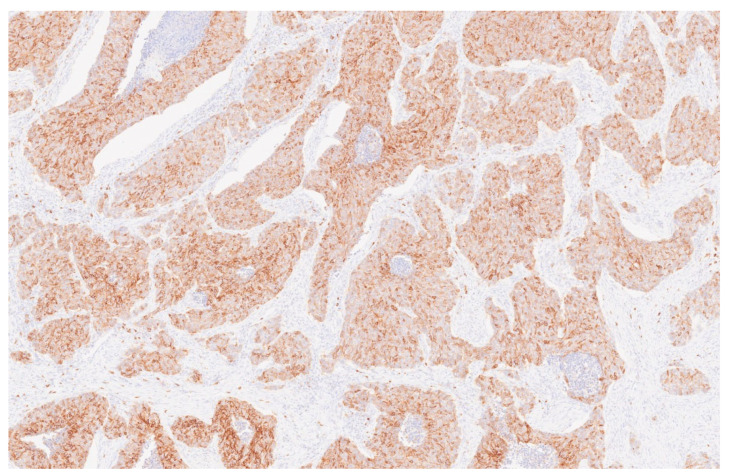
Adenoid cystic carcinoma section: Anti-CD117 antibody (7×).

**Immunohistochemical (IHC) Findings and Interpretation**: Immunohistochemical analysis demonstrated that the neoplastic cells were diffusely positive for pancytokeratins (AE1/AE3) and CK7, confirming the epithelial nature of the tumor. The neoplastic population also showed strong expression of Bcl-2 and CD117 (c-KIT), two markers commonly associated with adenoid cystic carcinoma (AdCC), thereby supporting the diagnosis (Figure 5 and Figure 6). Evaluation of myoepithelial differentiation, a key histological feature of AdCC, was performed using multiple markers. Although the tumor cells were negative for p63 and p40, myoepithelial cells were identified through positive staining for p63, p40, and calponin, confirming the presence of a biphasic (epithelial and myoepithelial) cellular component. Smooth muscle actin (SMA) showed only partial positivity, consistent with limited or focal expression, which is not uncommon in AdCC. The neoplastic cells were negative for MUC5, arguing against mucinous differentiation and helping to exclude mucin-producing adenocarcinomas from the differential diagnosis. **IHC Diagnostic Interpretation:** The immunophenotypic profile—characterized by epithelial marker expression (AE1/AE3, CK7), positivity for CD117 and Bcl-2, and the presence of myoepithelial cells identified by p63, p40, and calponin—was highly consistent with adenoid cystic carcinoma of the larynx. The lack of diffuse myoepithelial marker expression in the tumor cell population did not preclude the diagnosis, as the representation of myoepithelial cells can be variable or limited to specific tumor regions in AdCC.

**Figure 7:**
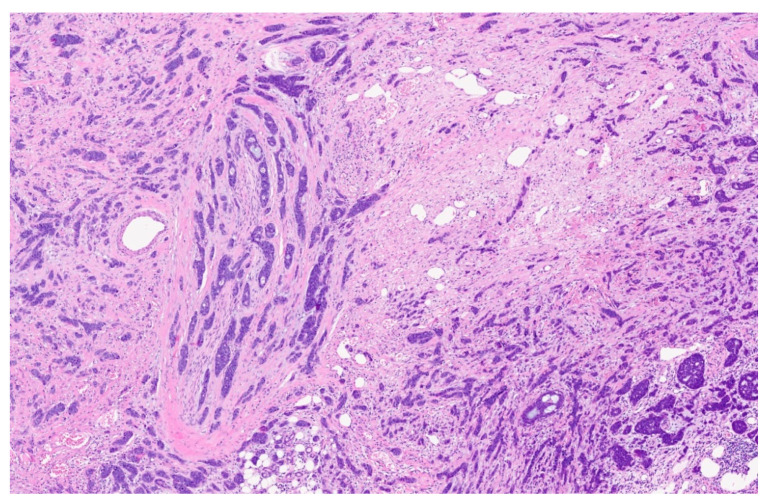
Perineural invasion (7×).

**Pathological Findings:** Histological examination of the laryngectomy specimen revealed a high-grade, poorly differentiated carcinoma consistent with the solid variant of adenoid cystic carcinoma (AdCC). The tumor involved both true vocal cords, anterior commissure, left ventricular band, and the left vocal muscle. It displayed predominantly solid and comedonic growth patterns, with extensive perineural invasion (Figure 7) and endolymphatic tumor emboli. Resection margins were negative, and no lymph node metastases were identified in bilateral selective neck dissections. These features, in conjunction with the immunohistochemical profile, confirmed the diagnosis of solid-type AdCC with aggressive pathological behavior. **Staging and Outcome:** According to the 2017 Union for International Cancer Control (UICC) classification, the tumor was staged as G3 pT3 pN0 V0 L1 Pn1 R0. **Discussion and Follow-Up:** This case is consistent with the existing literature on the clinical course and histological features of adenoid cystic carcinoma. In line with ASCO guidelines (2021) and given the tumor’s high grade and perineural invasion, adjuvant postoperative radiotherapy was recommended. Unfortunately, the patient died of a myocardial infarction a few days before radiotherapy could be initiated. Considering the rarity and biologically aggressive nature of adenoid cystic carcinoma of the larynx, alongside the patient’s advanced age and clinical presentation, a comprehensive surgical strategy including bilateral neck dissection was pursued to optimize local disease control and address the possibility of nodal involvement. Nonetheless, it is recognized that, in retrospect, the extent of the neck dissection may have been more extensive than what is typically indicated for this tumor subtype, particularly given the absence of histologically confirmed lymph node metastases. This case thus exemplifies the inherent challenge in balancing oncologic thoroughness with the minimization of treatment-related morbidity in rare head and neck malignancies, emphasizing the critical role of individualized, multidisciplinary clinical decision-making.

## 3. Discussion

Adenoid cystic carcinoma (ACC) of the larynx, although rare, presents unique diagnostic and therapeutic challenges due to its distinct biological behavior and anatomical constraints. While the general epidemiological features of laryngeal ACC are well documented [1,4], the present case highlights specific aspects that warrant further attention. Unlike many reports focusing on typical presentations and outcomes, this paper seeks to highlight the critical role of a multidisciplinary approach in the management of adenoid cystic carcinoma of the larynx, given its insidious nature.

Histologically, the cribriform subtype is most commonly reported and is typically associated with a more favorable prognosis, whereas the solid subtype is less frequent and generally linked to worse outcomes [1,4,46,47]. Histological subtype has to be considered as an important prognostic factor when dealing with ACC [48]. However, several authors have questioned the prognostic significance of histological subtype alone, suggesting that factors such as perineural invasion or tumor stage may confound these associations [46,47]. Our case supports the view that histology should not be considered in isolation when evaluating prognosis. This reflects the need for a more integrated assessment of pathological and clinical variables.

The subglottic region is consistently identified as the most common tumor location in laryngeal ACC, likely due to the distribution of minor salivary glands in this area [1,4,5,11]. The anatomical location—particularly the subglottic site, which is less accessible during routine examination—might also contribute to delayed diagnosis [18,49,50]. Our case reinforces this notion, aligning with previous reports.

Although a 5-year survival rate of approximately 70% has been reported [1,51], recurrence and metastasis remain common. Complete surgical excision with wide margins remains the primary therapeutic approach [8,9,12], yet the tumor’s propensity for submucosal and perineural spread frequently complicates resection, even in early-stage disease [8,9,12,22,52,53]. In this context, our findings are consistent with other reports that emphasize the challenges of achieving clear margins and long-term local control. Given the low incidence of lymph node metastases within the neck (6–10%) it is considered not always necessary to perform a neck dissection, reserving it for those cases where there is clinical or radiological evidence of lymph node metastases within the neck, although dissection of the paratracheal nodes is mandatory in case of subglottic the adenoid cystic carcinoma of the larynx [5,12,16,22,54].

The role of adjuvant radiotherapy, once controversial due to perceived radioresistance, is increasingly supported in the literature, particularly for cases involving high-grade histology, positive margins, or perineural invasion [4,8,9,12,55,56,57]. When carrying out radiotherapy treatment, some authors suggest administering at least 60Gy [10,12,55,56,57]. In our case, unfortunately adjuvant radiotherapy could not be administered. However, the lack of standardized radiation protocols and the limited role of chemotherapy—used mainly in palliative settings [1,4,47]—highlight the ongoing need for therapeutic innovation. Preliminary results involving targeted therapies such as lenvatinib [58,59] are promising, though further validation is required.

Post-treatment surveillance remains inconsistent across studies, with no consensus on follow-up protocols despite high recurrence rates. Furthermore, a noteworthy recent development is the transition from the 8th to the 9th edition of the AJCC TNM staging system, which excludes the subjective criterion of “extraparenchymal extension” in favor of more objective parameters such as tumor size and specific anatomical involvement [60,61]. Staging under the revised criteria has clear implications for prognosis and management. This aligns with recent proposals advocating for a tailored staging system for minor salivary gland tumors, which would better reflect their distinct patterns of spread and biological behavior [62].

In conclusion, this case adds to the current literature in several meaningful ways. Firstly, it presents a rare instance of the solid variant of adenoid cystic carcinoma (ACC) of the larynx—one of the least common histological subtypes—associated with a poorer prognosis and still underrepresented in contemporary reports. Secondly, the detailed “multimodal diagnostic workup”, particularly the integration of high-resolution imaging and comprehensive immunohistochemical profiling, provides a robust model for diagnosing rare laryngeal tumors with overlapping features. Thirdly, this report underscores the importance of a multidisciplinary tumor board approach in guiding evidence-based treatment decisions for rare malignancies, highlighting real-time clinical decision-making that incorporated findings from the most recent literature. Finally, the case also illustrates the clinical urgency and fragility involved in managing elderly patients with high-risk malignancies—despite curative surgery and a planned course of adjuvant therapy, the patient succumbed to an unrelated comorbidity (myocardial infarction), emphasizing the necessity of timely yet holistic care planning. As such, this case does not merely reiterate known features of laryngeal ACC but adds unique insights into the diagnostic, therapeutic, and systemic challenges associated with the solid subtype of laryngeal ACC in elderly patients, underlining the need for refined prognostic tools and integrated care strategies.

## 4. Conclusions

This case of adenoid cystic carcinoma (ACC) of the larynx illustrates the diagnostic and therapeutic challenges associated with this rare malignancy and offers several clinically significant insights. While the broader discussion reflects established knowledge, specific aspects of this presentation underscore important learning points.

Foremost, the histopathological diagnosis of the solid variant of ACC—recognized as the least favorable subtype—carries critical prognostic implications. The predominance of solid and comedonic patterns, as defined by the 2017 WHO classification, is associated with higher-grade biological behavior, an increased risk of recurrence, and poorer overall outcomes. This case reinforces the emerging consensus that histological subtype must be integrated into prognostic evaluation, particularly when coexisting with high-risk features such as perineural and lymphovascular invasion. Although some studies have questioned the independent prognostic value of histologic pattern alone, our findings support its essential role when interpreted in conjunction with other pathological variables. Furthermore, this case underscores the importance of a multidisciplinary approach to the management of laryngeal ACC, given its tendency for submucosal infiltration, perineural spread, and anatomical constraints that complicate oncologic resection. The decision to proceed with total laryngectomy and bilateral selective neck dissection followed a thorough interdisciplinary evaluation and aligned with current best practices for high-grade, locally advanced tumors. A significant limitation of this case, however, lies in the unexpected death of the patient due to myocardial infarction prior to the initiation of adjuvant radiotherapy—a treatment recommended due to high-risk histopathological features. This event prevented the completion of definitive therapy and highlights the importance of comprehensive pre-treatment assessment and the early management of comorbid conditions, particularly in elderly oncologic patients. The inability to evaluate the impact of radiotherapy in this context limits definitive conclusions regarding long-term disease control and overall prognosis. In summary, this case contributes to the limited literature on laryngeal ACC by highlighting the prognostic significance of histological subtype, particularly the solid variant, and by demonstrating the potential consequences of delayed or incomplete adjuvant therapy. It reinforces the critical role of individualized, multidisciplinary care and underscores the importance of addressing patient-specific factors that may influence treatment outcomes in head and neck oncology.

## Figures and Tables

**Table 1:** Flow diagram on laryngeal ACC management.

1	First assessment with endoscopic evaluation
2	Radiological investigation
3	Microlaryngoscopy with biopsy
4	Multidisciplinary tumor board's discussion
5	Treatment
6	Follow-up

**Table 2:** Available literature about ACC of larynx, published within the last ten years on PubMed; research keyword: “adenoid cystic carcinoma of larynx”.

	Author’s Name	Year of Publication	N. of Cases
Adenoid cystic carcinoma of the larynx: A report of two cases [19]	Guanqiao Li et al.	2015	2
Laryngeal adenoid cystic carcinoma: A population-based perspective [20]	Pariket M Dubal et al.	2015	69
Head and neck adenoid cystic carcinoma: A prospective multicenter REFCOR study of 95 cases [21]	M. Meyers et al.	2015	5
Laryngeal Adenoid Cystic Carcinoma: A Systematic Review [8].	Marchiano E. et al.	2016	120
Cervical Lymph Node Metastasis in Adenoid Cystic Carcinoma of the Larynx: A Collective International Review [22]	Andrés Coca-Pelaz et al.	2016	142
Malignant salivary gland tumours of the larynx: a single institution review [23]	S Karatayli-Ozgursoy et al.	2016	3
Adenoid cystic carcinoma of the larynx presenting with unusual subglottic mass: Case report [24]	Takashi Kashiwagi et al.	2016	1
Adenoid Cystic Carcinoma of the Larynx Presenting as a Thyroid Mass and Brief Literature Review [25]	Sadegh Shirian et al.	2017	1
Transoral Robotic Surgery Total Laryngectomy: Evaluation of Functional and Survival Outcomes in a Retrospective Case Series at a Single Institution [26]	Giri Krishnan et. al	2017	1
Adenoid cystic carcinoma of the larynx: a case report	L G Kozhanov et al.	2018	1
Adenoid cystic carcinoma of the larynx in a 70-year-old patient: A case report [1]	Filippo Ricciardiello et al.	2018	1
Adenoid cystic carcinoma in ventricle of larynx: An interesting case. [27]	Wang Q et al.	2018	1
The role of organ-and function-preserving radiotherapy in the treatment of adenoid cystic carcinoma of the larynx [28]	Sati Akbaba et al.	2019	11
The role of elective neck dissection in patients with adenoid cystic carcinoma of the head and neck [29]	Roy Xiao BA et al.	2019	51
Rare location of head and neck adenoid cystic carcinoma. [30]	Naim A et al.	2019	1
Minor salivary gland tumors of the head and neck-Memorial Sloan Kettering experience: Incidence and outcomes by site and histological type. [31]	Hay AJ et al.	2019	4
Laryngeal adenoid cystic carcinoma: Three cases reports. [32]	Cui Y et al.	2019	3
Endolaryngeal resection of the larynx for adenocystic cancer [33]	A L Kozhanov et al.	2019	1
Laryngeal adenoid cystic carcinoma: Radical or conservative surgery? [12]	Marco Lionello et al.	2021	17
Surgical management and oncological outcome of non-squamous cell carcinoma of the larynx: a bicentric study [34]	Andrea Iandelli et al.	2021	2
The glottic-subglottic laryngectomy: Surgical technique, oncological, and functional outcomes [35]	Andy Bertolin et al.	2022	9
Laryngeal adenoid cystic carcinoma: a rare case report. [36]	Eslami Aliabadi H et al.	2022	1
Four Years of Disease-Free Survival After Conservative Treatment of Subglottic Adenoid Cystic Carcinoma. [37]	Vardaxi C et al.	2022	1
Cricotracheal Adenoid Cystic Carcinoma: Insights Into the Diagnosis and Management of an Uncommon Anatomic Variant. [38]	Pacheco-Ojeda L et al.	2022	1
Adenoid Cystic Carcinoma of the Larynx: A SEER Database Review. [18]	Taha Mur et al.	2022	89
Fatal Tracheoesophageal Puncture Leakage Associated With Lenvatinib. [39]	Salvatori S et al.	2023	1
Malignant Minor Salivary gland neoplasms of Larynx: Our Experience. [40]	Joshi P et al.	2023	9
Successful surgery with preservation of laryngeal function in a patient with collision primary squamous cell carcinoma and adenoid cystic carcinoma in the hypopharynx and synchronous esophageal carcinoma: A case report [41]	Fu Z et al.	2023	1
Treatment and outcomes of minor salivary gland cancers of the larynx and trachea: a systematic review. [42]	Montenegro C et al.	2023	126
Subglottic Adenoid Cystic Carcinoma Mimicking Bronchial Asthma: A Case Report [43]	Athish KK et al.	2024	1
Fluoro-2-Deoxyglucose (FDG)-Avid Adenoid Cystic Carcinoma of the Larynx: A Rare Case and Diagnostic Insight Obtained Using Positron Emission Tomography/Computed Tomography (PET/CT) Imaging. [44]	Moghrabi S	2025	1
Adenoid cystic carcinoma of the larynx: Case report and review of literature. [45]	Es-Sahli FZ et al.	2025	1

## Data Availability

The original contributions presented in this study are included in the article. Further inquiries can be directed to the corresponding author.
